# Digital transformation project risks assessment using hybrid picture fuzzy distance measure-based additive ratio assessment method

**DOI:** 10.1038/s41598-025-86598-4

**Published:** 2025-01-26

**Authors:** Pratibha Rani, Arunodaya Raj Mishra, Adel Fahad Alrasheedi, Dragan Pamucar, Dragan Marinkovic

**Affiliations:** 1 Saveetha School of Engineering, Saveetha Institute of Medical and Technical Sciences (SIMATS), Chennai, Tamil Nadu India; 2 Department of Mathematics, Government College Raigaon, Satna, Madhya Pradesh 485441 India; 3 Department of Statistics and Operations Research, College of Science, King Saud University, 11451 Riyadh, Saudi Arabia; 4 Széchenyi István University, Győr, Hungary; 5 Institute of Mechanical Science, Vilnius Gediminas Technical University, Vilnius, 10105 Lithuania

**Keywords:** Picture fuzzy set, Distance measure, Digital transformation, Risks, Rank comparison (RANCOM), Additive ratio assessment (ARAS), Applied mathematics, Mathematics and computing

## Abstract

Digital transformation (DT) has become vital for companies trying to remain competitive in the recent ever-changing technological environment. DT is the integration of digital technologies into all disciplines of business from regular activities to strategic decision making. Risk management planning requires projects to assess possible risks that may negatively or positively affect a DT project. The purpose of the study is to introduce a hybridized decision support system (DSS) by combining the distance measure, ranking comparison (RANCOM) model and additive ratio assessment (ARAS) approach in the context of a picture fuzzy set (PFS). In this framework, the decision experts’ significance values are computed using a picture fuzzy score function-based formula. With the combination of objective weight using distance measure and subjective weight through the RANCOM model, a combined weight-determining approach is developed to determine the significance values of considered DT risks under picture fuzzy environment, while a hybrid ARAS model is developed to evaluate and rank DT projects from the risks perspective. To exhibit the feasibility of the introduced framework, a case study of a DT projects assessment problem is discussed in the context of picture fuzzy sets. A sensitivity study is also discussed over different values of the strategy coefficient, which confirms the strength of the proposed model. Further, a comparison with the existing picture fuzzy information-based methods is presented to prove the robustness of the developed decision-making framework.

## Introduction

Digital technologies have led to profound variations in companies’ activities including production, operations, management and sales methodologies^1^. Digital transformation (DT) is a procedure of embedding pioneering technologies into an organization’s products, processes and strategies. It forces companies to do major improvements in their business models and adapt to the new market reality. DT has gone from being a hi-tech opportunity to a pure requirement for handling the needs and potentials of the world’s increasing population^2^. DT and its related innovation have primarily transformed consumers’ expectations and behaviors, overstretched outdated firms, and interrupted several markets.

Companies inhabit the “main position” in relation to the industrial digitization and possess the competence to fruitfully grab the development visions resulting from the structure of the digital economy (DE) and DT. DT in any sector is not only related to firms’ strategic growth, persistence, and downfall but it also determines whether companies can reach new heights and grasp new prospects in the DE period^3,4^. In the recent past, many firms and even governments have introduced strategic foresight in education concerning the effect of DT^5,6^. There are great prospects in the direction of DT projects, however, the companies might face difficulties while planning to execute them^7^. Based on the existing studies, one of key complications to an effective development in DT projects is the lack of knowledge and uncertainty about DT, which often includes overlooked risks accompanying DT projects.

DT projects are risky and difficult due to the involvement of multiple tangible and intangible criteria. Multi-criteria decision-making (MCDM) models can be successfully applied to handle the DT project risk assessment problem. During the assessment of a DT project risks, the available information/data regarding diverse tangible and intangible criteria may be ambiguous or vague in nature. The concept of a fuzzy set (FS)^8^, which is determined uniquely with the membership degree (MD), has been widely used to handle the uncertainty of real decision-making problems. In the current literature, several scholars have labelled the generalizations as non-standard FSs^9,10^. One such generalization is an intuitionistic fuzzy set (IFS), initiated by Atanassov^11^, which includes non-membership degree (NMD) of an object to a FS with the constraint that the sum of MD and NMD should be ≤ 1. Though IFSs are more elegant than FSs in illustrating uncertain and ambiguous data, they lack a significant aspect, i.e., neutrality degree (ND), which plays a crucial role in several settings, namely, medical diagnosis, personal selection, and others^12^. For example, in medical diagnosis, symptoms such as temperature and body pain may have an insignificant effect on diseases compared with chest problems and stomach pain. Thus, to treat these issues, it is necessary to apply a new extension of FSs and IFSs, known as the picture fuzzy set (PFS)^13^. The PFS has been introduced by Cuong^13^, and in it each element is characterized by the positive, neutrality and negative membership degree, all belonging to [0, 1], whose collective sum is no more than 1. A key advantage of PFS is the inclusion of a “neutrality” degree, enriching the framework for refined MCDM, where a “maybe” or “neutral” attitude is relevant. With the use of picture fuzzy (PF) Kullback-Leibler divergence measure, Khattri et al.^14^ designed a clustering technique to address the problem of noise, vagueness, and non-linear structure present in an image. Some experiments have been provided for several image datasets and two publicly available brain magnetic resonance imaging datasets. Cao^15^ studied a conversion formula for picture fuzzy numbers (PFNs) and their corresponding right-angled triangular-based pyramids fuzzy numbers. Moreover, a similarity measure between PFSs has been introduced with its application in multimedia mobile healthcare services assessments. Ganie^16^ proposed a measure of PF-distance and applied it to solve pattern recognition problems. Using the PFSs-based MCDM approach, Mishra et al.^17^ assessed the drivers of DT in higher education institutes in which the information about the criteria and alternatives were given in terms of PFNs.

The classical ARAS model was introduced by Zavadskas and Turskis^18^ to prioritize the alternatives based on several criteria. This approach relies on the concept that the phenomena of complex domains with conflicting attributes could be dealt with by a simple relative comparison. This approach includes an intuitive and straightforward process that produces sensible, suitable and relatively precise outcomes in the assessments of different options with their performance parameters that are put forward as weighted evaluating attributes. It also provides a notion of an optimality rating of an option to obtain a preference for an option^18^. Soltani and Aliabadi^19^ presented a combined fuzzy SWARA-ARAS method for assessing the risks in firefighting. Mishra et al.^20^ integrated the ARAS method with dual probabilistic linguistic term sets and applied it to evaluate equipment suppliers in the medical field. In the context of PFSs, Jovčić et al.^21^ and Fan et al.^22^ developed the ARAS methods for different purposes, however, both these methods ignore the significance of DE weights as well as combined objective-subjective weights of criteria. To the best of our knowledge, there is no study that presents an integrated PF-DM-RANCOM-ARAS method for DT project risks assessment.

The determination of attribute weight is one of the more significant aspects of MCDM problems. In the literature, criteria weighting approaches are divided into two categories: objective and subjective^23^. In an objective weighting approach, the weight of criteria is estimated based on an aggregated decision-matrix with mathematical procedures without considering DE preferences, whereas in a subjective weighting tool, a criteria weight is computed from the DE opinion^24^ and assessment ratings. Considering objective weighting approaches, different entropy formulas are applied to determine weights, and this weighting method is invalid in various circumstances, thus it should be enhanced^25^. Here, we have developed a distance measure/cross entropy-based procedure to determine the weight of attributes. The criterion that has least entropy and large cross entropy should be allocated a large weight. Due to its effectiveness and innovation, we use the distance measure/cross entropy-based approach to determine objective weights of criteria, while subjective weights are estimated using RANking COMparison (RANCOM), which has first been developed by Więckowski et al.^26^ and preserves the pairwise comparison logic procedure using a three-value scale. RANCOM is developed for less experienced DEs and is characterized by robustness to variations in attribute associations, intuitiveness, time-efficiency in dealing with diverse MCDM problems, and effectiveness in handling imprecisions in assessments of different DEs while certifying high repeatability of outcomes. The approach includes finding criterion preferences, constructing a MAtrix of ranking Comparison (MAC) using pairwise assessments, estimating Summed Criteria Weights (SCW), and originating the overall weight of attributes.

According to current research, we find the following research challenges:

- In a recent study, Gölcük^27^ used an interval type-2 set-based model for evaluating risks of a DT project. This method cannot compute the significance of DE opinions. Moreover, it neglects the combined benefits of objective and subjective weights of risks. Furthermore, no article addresses the DT project risks assessment with picture fuzzy data.
- The determination of the criteria significance from DEs in an MCDM problem could be an interesting task. It is vital to develop a simple and intuitive approach that replicates the knowledge of DEs effectually. When an MCDM problem comprises various criteria, it is likely that an error will ensue in estimating criteria weight. Nevertheless, most subjective weight determining approaches function appropriately when DEs do not hesitate, are repeatable, and are very accurate. Thus, there is a need to improve formerly developed models to tackle DE hesitancy more efficiently. To fill this gap, it is necessary to propose a new approach, which can be applied to estimate criteria weight based on the information from DEs.
- Jovčić et al.^21^ presented a picture fuzzy extension of the ARAS method, while Fan et al.^22^ combined the ARAS and VIKOR approaches within the context of PFSs. These techniques used objective weights of criteria, which may reduce the bias coming from human opinions, however, it may concurrently overlook the opinions of DEs. Moreover, no extended version of the ARAS approach considers DE significance values during the assessment of MCDM problems with PFSs.

Considering the advantages of PFSs, this study develops a hybrid picture fuzzy MCDM methodology for assessing DT project risks. In this method, DE weights are computed through a PF-score function-based procedure, while the criteria weights are derived via a combined weighting formula consisting of objective and subjective weights by a PF-distance measure (PFDM)-based model and a PF-ranking comparison (RANCOM) model, respectively. To this aim, a modified picture fuzzy distance measure is proposed to quantify the degree of distance between PFSs. Finally, a modified picture fuzzy additive ratio assessment (ARAS) method is proposed to rank the DT project risks.

The prime objectives of this study are as follows:

- A new distance measure is proposed to compute the degree of difference between PFSs.
- An approach for finding the weight of criteria is presented based on the objective and subjective weights through PFDM and PF-RANCOM models, respectively.
- A hybrid picture fuzzy ARAS method is introduced in which the information about the criteria and DEs is fully unknown.
- The developed ARAS approach is applied to a case study of a DT project risks assessment, which shows its pertinence and feasibility.
- Sensitivity and comparative discussions are made to reveal the strength and steadiness of the introduced model.

The rest of the paper is structured in the following manner: Section “Related works” confers the comprehensive works related to DT projects. Section “Problem definition” describes the alternatives and criteria. Section “A hybrid PF-DM-RANCOM-ARAS method for solving MCDM problems” first presents the basic definitions, then develops a PFDM, and lastly proposes a hybrid PF-DM-RANCOM-ARAS methodology for handling MCDM problems. Section “Case study: assessment of DT project risks” implements the developed method to a case study of DT project risks evaluation. Section “Sensitivity and comparative study discussion” presents the results and discussion. Section “Conclusions” concludes the study and recommends the scope for future research.

## Related works

Recently, DT has appeared as a significant phenomenon in strategic business activities. It has become an indispensable aspect of modern society, profoundly affecting organizations in various sectors. It offers new challenges and opportunities for all industries and the organizations cooperating in industrial networks. DT has been described in many ways, and several studies on this topic have been concentrated on identifying technological aspects and capabilities. For instance, Bruskin et al.^28^ presented realistic implications of development of sustenance structures for digital corporate organizations and delineated the opportunities. By means of an incorporated system architecture, Masuda et al.^29^ investigated DT risks in firm design areas and explained risk mitigation stratagems by suggesting a unified scheme architecture. With the AHP tool, Yoo and Kim^30^ explored the critical factors in order to adopt cloud computing. Jayakrishnan et al.^31^ analyzed the digitalization models in the Malaysian transportation sector. Elezaj et al.^32^ recapitulated and studied the big data (BD) initiatives of e-government operated in diverse nations. Fritzsche et al.^33^ exposed the modifications between reports of intergovernmental enterprises and pertinent literature to reveal the association with climate change and DT. Young and Rogers^7^ presented a comprehensive analysis comprising the foundation of DT’s mechanisms with specific importance for the mining sector. Battisti et al.^34^ presented the relationship between risk management and BD with a specific situation in the commercial estates. Ricciardi et al.^35^ inspected DT in healthcare facilities and further emphasized the impact of critical factors for evaluating and observing DT projects. Ramos et al.^36^ developed an assessment model combining the AHP and PROMETHEE models and applied it to evaluate the requirements for industry 4.0 (I4.0). Jones^37^ highlighted the character of DT on the procedure of safety in enterprises. Eckhart et al.^38^ pointed out the significant problems and challenges occurring in quantifiable security risks evaluation for industrialized control structures. Mitra and O’Regan^39^ explored the role of inventive management in cyber asset marketplaces.

Further, Scholz et al.^40^ developed a pioneering technique for handling organizational vulnerability and resilience management. Saarikko et al.^41^ used IoT as a milieu to validate the issues related to transformative technologies. Further, they presented five recommendations to show how companies can propose the policies required for DT and become digitally sensible. Gölcük^27^ proposed a risk evaluation tool by uniting the best worst model with interval type-2 fuzzy information perspective. Liu et al.^3^ applied the text mining approach to create an index of DT and illustrate the impact of DT on enterprise innovation and its scheme from theoretical and empirical viewpoints. They studied whether DT can meaningfully improve firms’ worth with the innovation. Mishra et al.^17^ used a PFSs-based method to assess the drivers of DT in higher education institutions. However, there is no study which assesses DT project risks within the context of PFSs, even though the PFS theory is an efficient way to tackle the uncertainty of realistic problems.

Motivated by the concerns of extant literature, this paper introduces a hybrid PF-DM-RANCOM-ARAS model to assess DT projects from the risks perspective. The developed model does not only assess the considered DT project options through the developed PF-DM-RANCOM-ARAS method, but also computes the weight values of DT risks and DEs. The developed approach can help DEs to obtain more assured findings in the assessment of DT project risks.

## Problem definition

**Fig. 1:**
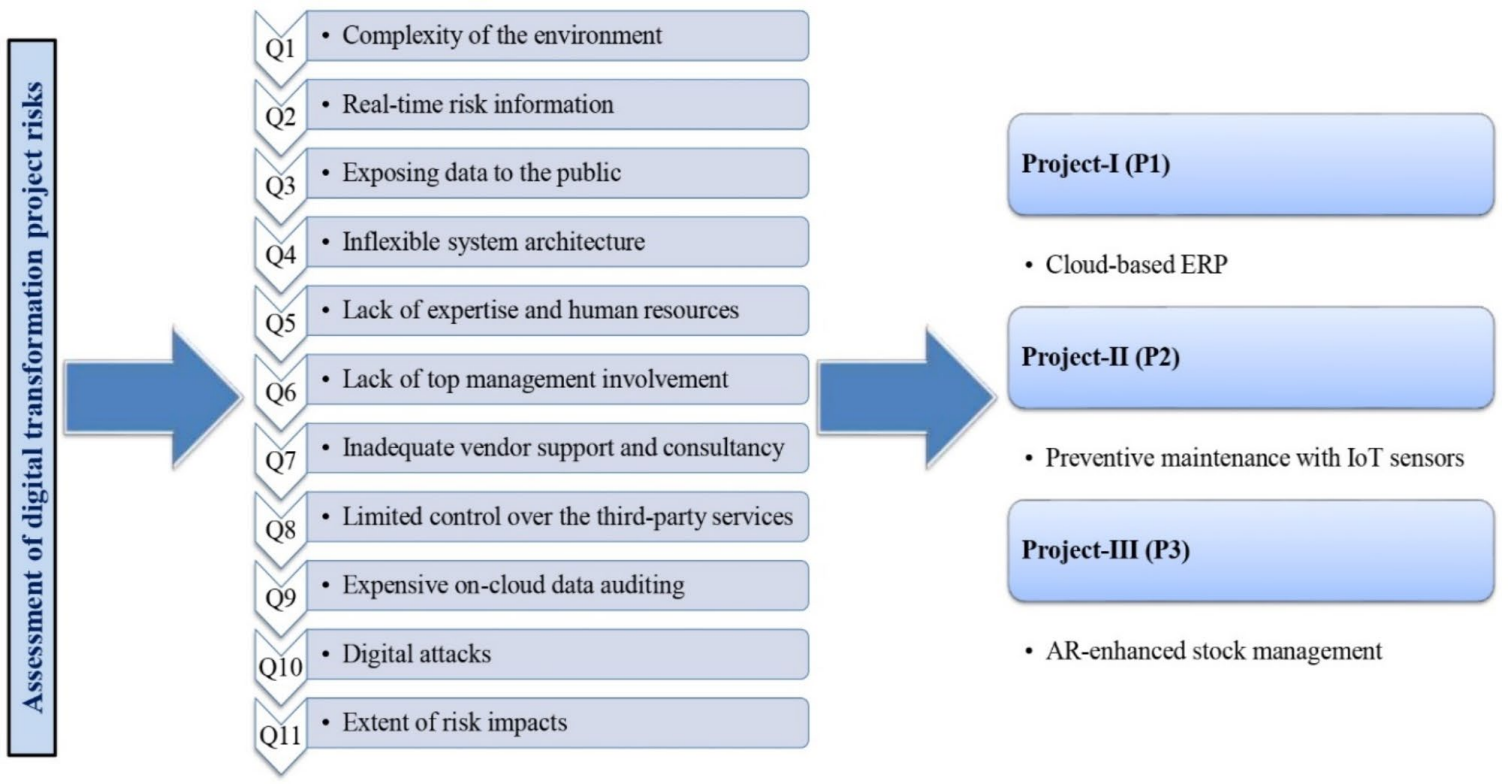
Description of criteria and options for DT project risks assessment.

DT is the sociocultural procedure of transforming businesses to innovative structural forms and skill sets required to endure feasible and pertinent risks in the DE era^41^. In general, the term ‘DT’ may be defined as a “process that purposes to enhance an object by triggering important changes to its properties with the associations of information, computing, communication, and connectivity technologies”^41^. Owing to the presence of numerous risks, identifying and evaluating DT projects can be considered as a challenging and uncertain MCDM problem. In this study, a risk assessment framework is developed. The novel risk assessment framework comprises the problem defining stage, risk measurement stage, and development of an MCDM model stage. The risk measurement stage implements a general procedure of considering risk factors (RFs), risk likelihood (RL) and risk severity (RS)^27,42^. The relative significance (or weight) of each RF is obtained using the developed PF-distance measure-RANCOM approach. Risk magnitudes (RMs) are determined using a PF information-based system. All of the MDs are assumed to be PFNs. Figure 1 shows the graphical description of DT projects as options and risks as criteria. In what follows, DEs are recognized and problems (projects) are elucidated. Risk factors are considered with thorough literature analyses and DE decisions. Further, risk evaluation attributes, presented in Table 1, are estimated to implement the study. For determining the linguistic values (LVs), an eight-point Likert scale is used, as shown in Table 2. The different LVs that will be utilized in diverse stages of assessments are obtained. Afterwards, the PFNs associated with the LVs of different risk factors and DT projects are identified for assessing DT project risks.

**Table 1:** Considered criteria for DT project risks assessment.

Risks	References
Complexity (*Q*_1_)	^37,38,43,44^
Real-time risk information (*Q*_2_)	^37^
Exposing data to the public (*Q*_3_)	^27,45^
Inflexible system architecture (*Q*_4_)	^27^
Budget constraints (*Q*_5_)	^4^
Lack of digital mindset (*Q*_6_)	^27,29^
Ineffective vendor management (*Q*_7_)	^46^
Limited control over third-party services (*Q*_8_)	^27^
Lack of participation of top management (*Q*_9_)	^4,27^
Digital attacks (*Q*_10_)	^47^
Extent of risk impacts (*Q*_11_)	^37,43,44^

**Table 2:** Linguistic values and corresponding PFNs^17^.

LRs	PFNs
Very very high (VVH)	(0.9, 0.05, 0.05)
Very High (VH)	(0.8, 0.05, 0.1)
High (H)	(0.75, 0.1, 0.1)
Almost high (AH)	(0.6, 0.1, 0.2)
Average (A)	(0.5, 0.1, 0.4)
Low (L)	(0.3, 0.05, 0.6)
Quite low (QL)	(0.2, 0.05, 0.7)
Very low (VL)	(0.1, 0.05, 0.8)

### Description of DT Project options

In this section, we consider three DT projects: Oracle fusion cloud Enterprise Resource Planning software (*P*_1_), Augmented Reality-based warehouse management (*P*_2_), and Predictive maintenance with IoT sensors (*P*_3_). The description of the options is given as follows:

***Oracle fusion cloud enterprise resource planning software*** (***P***_***1***_) It is a latest complete cloud enterprise resource planning suite that manipulates all essential functions competently to perform proper business processes. It is a suite of tools for data storage and analysis in a cost-effective manner.

***Augmented reality-based warehouse management*** (***P***_***2***_) Augmented reality enhances the efficiency and accuracy of various data collection procedures and leads to effective warehouse management. It can simplify the supply chain procedure by empowering highly realistic 3D visualizations, real-time object tracking, and advanced stock and inventory management.

***IoT-based preventive maintenance*** (***P***_***3***_) It is a maintenance strategy that uses IoT to gather and analyze data about resources, equipment or technology. An IoT-based predictive maintenance system can help to predict possible damage by collecting data from ultrasonic and vibration sensors.

### Description of DT risks

In this part of the study, we will discuss the considered DT risks as follows:

***Complexity*** (***Q***_***1***_) The occurrence of new and extremely dynamic risk factors, high vitality of the environment, open system interconnection, and interconnection between exposures construct a complicated situation for the businesses in the context of DT^37,38,43,44^.

***Real-time risk information*** (***Q***_***2***_) As risks are addressed in diverse ways based on the role and MCDM levels in organizations, it is important that stockholders should know the risk information during DT projects management^37^.

***Data breach*** (***Q***_***3***_) It is a security incident that occurs when sensitive data is inadvertently visible to the public^45^. It is often caused by human error, negligence, poor data security and sanitization or malicious attack^27^.

***Inflexible system architecture*** (***Q***_***4***_) Flexible manufacturing systems require significant initial investment because they from the technical infrastructure of any business. If a project possesses an inflexible system architecture, it becomes problematic to respond to changing customer requirements^27^.

***Budget constraints*** (***Q***_***5***_) In the process of digitalization, assimilating new tools, training personnel, hiring digital experts and advisors will undeniably increase the costs of companies. Many businesses face funding issues during their DT planning^4^.

***Lack of digital mindset*** (***Q***_***6***_) Leadership in the digital age needs a modification of the mindset and aids in navigating the difficulties of DT. However, top leaders lack the mindset required to successfully drive DT project implementation initiatives^29^.

***Ineffective vendor management*** (***Q***_***7***_) Vendor management is one of the significant steps in a successful DT enterprise. Communication breakdowns and poor vendor relationships often cause misinterpretations, costly setbacks, inadequate service and worst-case scenarios^46^.

***Limited control over third-party services*** (***Q***_***8***_) It permits firms to integrate external data, namely programs, emails and documents, from multichannels^27^. However, breaches in third party-services cause transactional and security issues in businesses.

***Lack of participation of top management*** (***Q***_***9***_) For any technological improvement, top management should be involved and fully invested in the transformation. During the process of DT project implementation, the management should properly explain the process, advantages and disadvantages to the employees^4,27^.

***Digital attacks*** (***Q***_***10***_) It aims to evaluate, modify or destroy sensitive information, extract economic benefits from employers or interrupt usual business procedures. Fraudsters may take benefit of susceptibilities in digital technologies; thus, the management must certify that technological outcomes are secure from digital attacks^47^.

***Extent of risk impacts*** (***Q***_***11***_) Since organizational systems (OSs) are interconnected, risk factors derived from a particular structural subsystem can lead to prolonged influence on the entire business^43,44^. Considering and evaluating the aggregate risk impact supports better decision analysis from a risk management standpoint^37^.

## A hybrid PF-DM-RANCOM-ARAS method for solving MCDM problems

This section first presents the basic concepts of PFSs, followed by a proposed distance measure for PFSs. Moreover, this section introduces an integrated PF-DM-RANCOM-ARAS methodology for solving the DT project risk selection problem.

### Basic concepts

#### Definition 4.1

Cuong^13^. A PFS *D* on a fixed set $U=\left\{ {{q_1},{q_2},.,{q_m}} \right\}$ is mathematically expressed as

$$D=\left\{ {\left\langle {{q_i},\left( {{\mu _D}\left( {{q_i}} \right),{\eta _D}\left( {{q_i}} \right),{\nu _D}\left( {{q_i}} \right)} \right)} \right\rangle \left| {{q_i} \in U} \right.} \right\},$$

where ${\mu _D}({q_i}):U \to \left[ {0,1} \right]$ denotes positive, ${\eta _D}({q_i}):U \to \left[ {0,1} \right]$ denotes neutral and ${\nu _D}({q_i}):U \to \left[ {0,1} \right]$ denotes negative MDs of an element *q*_*i*_ in *D*, respectively, satisfying $0 \leq {\mu _D}\left( {{q_i}} \right)+{\eta _D}\left( {{q_i}} \right)+{\nu _D}\left( {{q_i}} \right) \leq 1.$ For each ${q_i} \in U,$ the refusal MD is defined as ${\rho _D}\left( {{q_i}} \right)=1 - \left( {{\mu _D}\left( {{q_i}} \right)+{\eta _D}\left( {{q_i}} \right)+{\nu _D}\left( {{q_i}} \right)} \right).$ For simplicity, the term “$\left( {{\mu _D},{\eta _D},{\nu _D}} \right)$” is defined as the ‘picture fuzzy number (PFN)’ and is symbolized as $\theta =\left( {\mu ,\eta ,\nu } \right).$

#### Definition 4.2

Wang et al.^48^ and Ju et al.^49^. For a PFN $\theta =\left( {\mu ,\eta ,\nu } \right),$ score and accuracy values are determined using Eqs. (1) and (2), respectively.

1 $${\mathbb{S}}\left( \theta \right)=\mu - \eta - \nu ,\quad\hbox{where}\quad {\mathbb{S}}\left( \theta \right) \in \left[ { - 1,1} \right],$$

2 $$A\left( \theta \right)=\mu +\eta +\nu,\quad\hbox{where}\quad A\left( \theta \right) \in \left[ {0,1} \right].$$

Further, Ju et al.^49^ defined the normalized score function on PFS as

3 $$\tilde {\mathbb{S}}\left( \theta \right)=\frac{1}{3}\left( {2+{\mathbb{S}}\left( \theta \right)} \right),\quad\hbox{where}\quad \tilde {\mathbb{S}}\left( \theta \right) \in \left[ {0,1} \right].$$

#### Definition 4.3

Cuong^13^. Let ${\theta _1}=\left( {{\mu _1},{\eta _1},{\nu _1}} \right)$ and ${\theta _2}=\left( {{\mu _2},{\eta _2},{\nu _2}} \right)$ be two PFNs. Some operations on PFNs are presented as follows:

(i) $$\theta _{k}^{c}=\left( {{\nu _k},{\eta _k},{\mu _k}} \right),\,k=1,2,$$
(ii) $${\theta _1} \subseteq {\theta _2}$$ iff $${\mu _1} \leq {\mu _2},$$$${\eta _1} \leq {\eta _2}$$ and $${\nu _1} \geq {\nu _2},$$
(iii) ${\theta _1}={\theta _2}$ iff ${\theta _1} \subseteq {\theta _2}$ and ${\theta _1} \supseteq {\theta _2},$
(iv) $${\theta _1} \oplus {\theta _2}=\left\{ {\left( {{\mu _1}+{\mu _2} - {\mu _1}{\mu _2},{\eta _1}{\eta _2},{\nu _1}{\nu _2}} \right)} \right\},$$
(v) $${\theta _1} \otimes {\theta _2}=\left\{ {\left( {{\mu _1}{\mu _2},{\eta _1}+{\eta _2} - {\eta _1}{\eta _2},{\nu _1}+{\nu _2} - {\nu _1}{\nu _2}} \right)} \right\}.$$

#### Definition 4.4

Dinh and Thao^50^. Let $D,E,F \in PFSs\left( U \right).$ A real-valued function $\Im :PFS(U) \times PFS(U)$ $\to [0,1]$ is said to be a picture fuzzy distance measure if it holds the following requirements:

(d_1_): $$0 \leq \Im (D,E) \leq 1,$$
(d_2_): $$\Im (D,E)=0$$ iff $$D=E,$$
(d_3_): $$\Im (D,E)=\Im (E,D),$$
(d_4_): If $D \subseteq E \subseteq F,$ then $\Im (D,E) \leq \Im (D,F)$ and $\Im (E,F) \leq \Im (D,F).$

### New distance measure for PFSs

For $D,E \in PFSs\left( U \right),$ we propose a new distance measure for PFSs, given as

4 $$\Im \left( {D,E} \right)=\frac{1}{m}\sum\limits_{{i=1}}^{m} {g\left( {\left| {{\mu _D}({q_i}) - {\mu _E}({q_i})} \right|,\left| {{\eta _D}({q_i}) - {\eta _E}({q_i})} \right|,\left| {{\nu _D}({q_i}) - {\nu _E}({q_i})} \right|} \right)} ,$$

where ‘$g$’ is a *t*-conorm.

#### Property 4.1

*For* $$D,E \in PFSs\left( U \right),$$ $$0 \leq \Im (D,E) \leq 1.$$

#### Proof

As $$D,E \in PFSs\left( U \right),$$ then by definition of PFS, we have $$0 \leq {\mu _D}({q_i})+{\eta _D}({q_i})+{\nu _D}({q_i}) \leq 1$$ and $$0 \leq {\mu _E}({q_i})$$$$+{\eta _E}({q_i})+{\nu _E}({q_i}) \leq 1,$$ $$\forall {q_i} \in U.$$ It implies that $$0 \leq \left| {{\mu _D}({q_i}) - {\mu _E}({q_i})} \right| \leq 1,$$$$0 \leq \left| {{\eta _D}({q_i}) - {\eta _E}({q_i})} \right| \leq 1$$ and $$0 \leq \left| {{\nu _D}({q_i}) - {\nu _E}({q_i})} \right| \leq 1,$$ $$\forall {q_i} \in U.$$ Hence, $$0 \leq \Im \left( {D,E} \right) \leq 1.$$

#### Property 4.2

*For* $$D,E \in PFSs\left( U \right),$$$$\Im (D,E)=0$$ *iff* $$D=E.$$

#### Proof

When $D=E,$ then it is obvious from Eq. (4) that $\Im \left( {D,E} \right)=0.$ On the other hand, when $\Im \left( {D,E} \right)=0,$ we have

$$\begin{aligned}\Im \left( {D,E} \right)&= \frac{1}{m}\sum\limits_{{i=1}}^{m} {g\left( {\left| {{\mu _D}({q_i}) - {\mu _E}({q_i})} \right|,\,\left| {{\eta _D}({q_i}) - {\eta _E}({q_i})} \right|,\,\left| {{\nu _D}({q_i}) - {\nu _E}({q_i})} \right|} \right)} =0,\\ &\Leftrightarrow g\left( {\left| {{\mu _D}({q_i}) - {\mu _E}({q_i})} \right|,\,\left| {{\eta _D}({q_i}) - {\eta _E}({q_i})} \right|,\,\left| {{\nu _D}({q_i}) - {\nu _E}({q_i})} \right|} \right)=0,\\ &\Leftrightarrow \left| {{\mu _D}({q_i}) - {\mu _E}({q_i})} \right|=0,\,\left| {{\eta _D}({q_i}) - {\eta _E}({q_i})} \right|=0\quad\hbox{and}\quad \left| {{\nu _D}({q_i}) - {\nu _E}({q_i})} \right|=0,\\ &\Leftrightarrow {\mu _D}({q_i})={\mu _E}({q_i}),\quad {\eta _D}({q_i})={\eta _E}({q_i})\quad\hbox{and}\quad {\nu _D}({q_i})={\nu _E}({q_i}),\\ &\Leftrightarrow D=E.\end{aligned}$$

#### Property 4.3

*For* $$D,E \in PFSs\left( U \right),$$$$\Im (D,E)=\Im (E,D).$$

#### *Proof*

$$\begin{aligned}\Im \left( {E,D} \right)&=\frac{1}{m}\sum\limits_{{i=1}}^{m} {g\left( {\left| {{\mu _E}({q_i}) - {\mu _D}({q_i})} \right|,\,\left| {{\eta _E}({q_i}) - {\eta _D}({q_i})} \right|,\,\left| {{\nu _E}({q_i}) - {\nu _D}({q_i})} \right|} \right)} \\ &=\frac{1}{m}\sum\limits_{{i=1}}^{m} {g\left( {\left| {{\mu _D}({q_i}) - {\mu _E}({q_i})} \right|,\,\left| {{\eta _D}({q_i}) - {\eta _E}({q_i})} \right|,\,\left| {{\nu _D}({q_i}) - {\nu _E}({q_i})} \right|} \right)} =\Im \left( {D,E} \right).\end{aligned}$$

#### Property 4.4

*If* $D \subseteq E \subseteq F,$ *then* $\Im (D,E) \leq \Im (D,F)$ *and* $\Im (E,F) \leq \Im (D,F),$ *where* $D,E,F$ $\in PFSs\left( U \right).$

#### Proof

For $$D \subseteq E \subseteq F,$$ we have $$\left| {{\mu _D}({q_i}) - {\mu _E}({q_i})} \right|$$ $$\leq \left| {{\mu _D}({q_i}) - {\mu _F}({q_i})} \right|$$, $$\left| {{\eta _D}({q_i}) - {\eta _E}({q_i})} \right|$$ $$\leq \left| {{\eta _D}({q_i}) - {\eta _F}({q_i})} \right|$$ and $$\left| {{\nu _D}({q_i}) - {\nu _E}({q_i})} \right|$$$$\leq \left| {{\nu _D}({q_i}) - {\nu _F}({q_i})} \right|$$, $$\forall {q_i} \in U.$$ In addition, $$\left| {{\mu _E}({q_i}) - {\mu _F}({q_i})} \right|$$$$\leq \left| {{\mu _D}({q_i}) - {\mu _F}({q_i})} \right|,$$$$\left| {{\eta _E}({q_i}) - {\eta _F}({q_i})} \right|$$$$\leq \left| {{\eta _D}({q_i}) - {\eta _F}({q_i})} \right|$$and $$\left| {{\nu _E}({q_i}) - {\nu _F}({q_i})} \right|$$$$\leq \left| {{\nu _D}({q_i}) - {\nu _F}({q_i})} \right|,\forall {q_i} \in U.$$ It implies that

$$\begin{aligned}&g\left( {\left| {{\mu _D}({q_i}) - {\mu _E}({q_i})} \right|,\,\left| {{\eta _D}({q_i}) - {\eta _E}({q_i})} \right|,\,\left| {{\nu _D}({q_i}) - {\nu _E}({q_i})} \right|} \right)\\ &\quad\leq g\left( {\left| {{\mu _D}({q_i}) - {\mu _F}({q_i})} \right|,\,\left| {{\eta _D}({q_i}) - {\eta _F}({q_i})} \right|,\,\left| {{\nu _D}({q_i}) - {\nu _F}({q_i})} \right|} \right)\end{aligned}$$

and

$$\begin{aligned}&g\left( {\left| {{\mu _E}({q_i}) - {\mu _F}({q_i})} \right|,\,\left| {{\eta _E}({q_i}) - {\eta _F}({q_i})} \right|,\,\left| {{\nu _E}({q_i}) - {\nu _F}({q_i})} \right|} \right)\\ &\quad\leq g\left( {\left| {{\mu _D}({q_i}) - {\mu _F}({q_i})} \right|,\,\left| {{\eta _D}({q_i}) - {\eta _F}({q_i})} \right|,\,\left| {{\nu _D}({q_i}) - {\nu _F}({q_i})} \right|} \right),\forall {q_i} \in U.\end{aligned}$$

Thus, we have $$\Im \left( {D,F} \right) \geq \Im \left( {D,E} \right)$$ and $$\Im \left( {D,F} \right) \geq \Im \left( {E,F} \right),$$$$\forall D,E,F \in PFSs\left( U \right).$$

#### Theorem 4.1

*For two PFSs* $D=\left\{ {\left\langle {{q_i},\,\left( {{\mu _D}\left( {{q_i}} \right),\,{\eta _D}\left( {{q_i}} \right),\,{\nu _D}\left( {{q_i}} \right)} \right)} \right\rangle \left| {{q_i} \in U} \right.} \right\}$ *and* $E=\{ \langle {q_i},\,( {\mu _E}( {{q_i}}),\,{\eta _E}( {{q_i}} ),$ ${\nu _E}( {{q_i}})) \rangle | {{q_i} \in U} \},$ *the function* $\Im \left( {D,E} \right)$ *is a distance measure for PFSs.*

#### Proof

It is clear from Properties 4.1–4.4.

#### Note

(i) If $$g\left( {\alpha ,\gamma } \right)=\hbox{min} \left\{ {1,\alpha +\gamma } \right\},$$ then

5 $${\Im _1}\left( {D,E} \right)=\frac{1}{m}\sum\limits_{{i=1}}^{m} {\hbox{min} \left( {1,\,\left| {{\mu _D}({q_i}) - {\mu _E}({q_i})} \right|+\left| {{\eta _D}({q_i}) - {\eta _E}({q_i})} \right|+\left| {{\nu _D}({q_i}) - {\nu _E}({q_i})} \right|} \right)} .$$

(ii) If $g\left( {\alpha ,\gamma } \right)=\alpha +\gamma - \alpha .\gamma ,$ then

6 $${\Im _2}\left( {D,E} \right)=\frac{1}{m}\sum\limits_{{i=1}}^{m} {\left[ \begin{aligned} \left| {{\mu _D}({q_i}) - {\mu _E}({q_i})} \right|+\left| {{\eta _D}({q_i}) - {\eta _E}({q_i})} \right|+\left| {{\nu _D}({q_i}) - {\nu _E}({q_i})} \right| \hfill \\ - \left| {{\mu _D}({q_i}) - {\mu _E}({q_i})} \right|.\left| {{\eta _D}({q_i}) - {\eta _E}({q_i})} \right|.\,\left| {{\nu _D}({q_i}) - {\nu _E}({q_i})} \right| \hfill \\ \end{aligned} \right]} .$$

(iii) If $g\left( {\alpha ,\gamma } \right)=\frac{{\alpha +\gamma - 2\alpha .\gamma }}{{1 - \alpha .\gamma }},$ then

7 $${\Im _3}\left( {D,E} \right)=\frac{1}{m}\sum\limits_{{i=1}}^{m} {\left[ {\frac{\begin{aligned} \left| {{\mu _D}({q_i}) - {\mu _E}({q_i})} \right|+\left| {{\eta _D}({q_i}) - {\eta _E}({q_i})} \right|+\left| {{\nu _D}({q_i}) - {\nu _E}({q_i})} \right| \hfill \\ - 2\left| {{\mu _D}({q_i}) - {\mu _E}({q_i})} \right|.\left| {{\eta _D}({q_i}) - {\eta _E}({q_i})} \right|.\,\left| {{\nu _D}({q_i}) - {\nu _E}({q_i})} \right| \hfill \\ \end{aligned} }{{1 - \left| {{\mu _D}({q_i}) - {\mu _E}({q_i})} \right|.\left| {{\eta _D}({q_i}) - {\eta _E}({q_i})} \right|.\,\left| {{\nu _D}({q_i}) - {\nu _E}({q_i})} \right|}}} \right]} .$$

#### Theorem 4.2

*Let* $D,E \in PFSs\left( U \right).$ *Then, for the proposed PFDM, Eq.* (4) *satisfies the following properties:*

(i) $$\Im \left( {{D^c},\,{E^c}} \right)=\Im \left( {D,E} \right),$$
(ii) $$\Im \left( {D,{E^c}} \right)=\Im \left( {{D^c},E} \right),$$
(iii) $$\Im \left( {D,{D^c}} \right)=0$$ *iff* $${\mu _D}\left( {{o_i}} \right)={\nu _D}\left( {{o_i}} \right),\forall {o_i} \in U,$$
(iv) $$\Im \left( {D \cap E,E} \right) \leq \Im \left( {D,E} \right),$$
(v) $$\Im \left( {D \cup E,E} \right) \leq \Im \left( {D,E} \right).$$

#### Proof

The proof of Theorem 4.2 follows the abovementioned Properties 4.1–4.4.

### A picture fuzzy extension of ARAS method

**Fig. 2:**
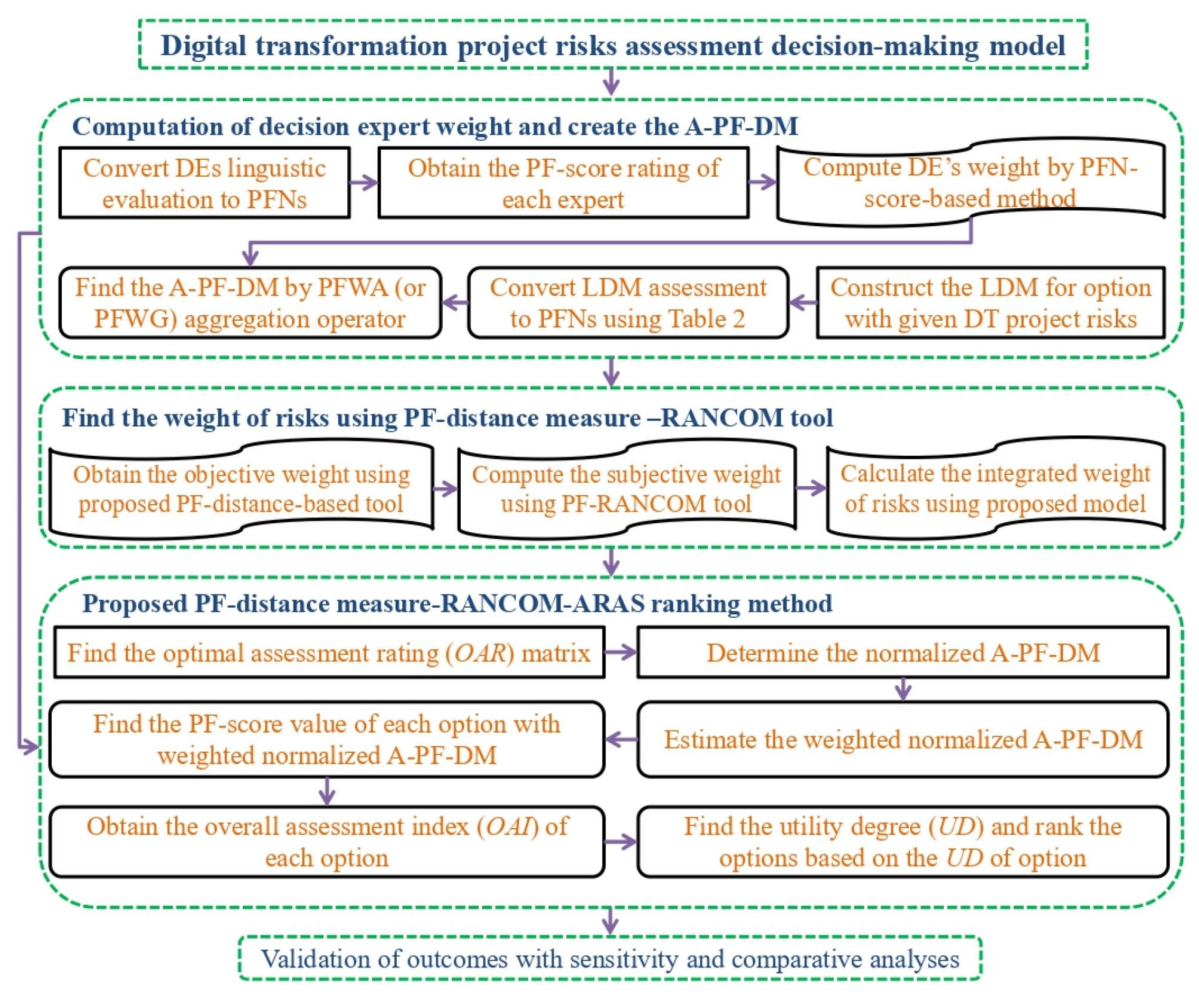
Graphical framework of the proposed PF-RANCOM-ARAS method.

In this subsection, we extend the classical ARAS method to a picture fuzzy environment based on the criteria and DE weighting models. Let us consider a set of alternatives $P=\left\{ {{P_1},{P_2},\ldots,{P_m}} \right\}$ which will be evaluated based on the criteria set $Q=\left\{ {{Q_1},{Q_2},\ldots,{Q_n}} \right\}.$ A group of DEs $B=\left\{ {{b_1},{b_2},\ldots,{b_r}} \right\}$ is invited to evaluate the options by means of the given criteria in terms of PFNs. Let $Y=\left( {y_{{ij}}^{{(l)}}} \right)$ be the linguistic matrix (LM) given by an expert committee, where $y_{{ij}}^{{(l)}}$ denotes the linguistic performance value of the *i*^th^ alternative concerning the *j*^th^ criterion, presented by the *l*^th^ DE based on Table 2. The procedural steps of the developed method are given as follows (see Fig. 2 for pictorial representation):

**Step 1** Derive the weights of DEs.

Let ${\lambda _l}=\left( {{\mu _l},{\eta _l},{\nu _l}} \right)$ be the picture fuzzy significance value of the *l*^th^ DE. We use the following formula to compute the numeric weight of the *l*^th^ DE:

8 $${\psi _l}=\frac{{\left( {{\mu _l} - {\eta _l} - {\nu _l}} \right)}}{{\sum\nolimits_{{l=1}}^{r} {\left( {{\mu _l} - {\eta _l} - {\nu _l}} \right)} }},\quad l=1,2,\ldots,r.$$

Here, ${\psi _l}$ should be non-negative and ${\psi _1}+{\psi _2}+\cdots+{\psi _r}=1.$

**Step 2** Aggregate the individual DE opinions.

In this step, the PFWA operator (Cuong, 2014) is used to create an aggregated picture fuzzy decision matrix (APF-DM) ${\mathbb{Z}}={\left( {{z_{ij}}} \right)_{m \times n}},$ where

9 $${z_{ij}}=\left( {{\mu _{ij}},\,{\eta _{ij}},{\nu _{ij}}} \right)=PFWA\left( {y_{{ij}}^{{\left( 1 \right)}},y_{{ij}}^{{\left( 2 \right)}},\ldots,y_{{ij}}^{{\left( r \right)}}} \right)=\left( {1 - \prod\limits_{{l=1}}^{r} {{{(1 - {\mu _l})}^{{\psi _l}}},\,\prod\limits_{{l=1}}^{r} {({\eta _l}} } {)^{{\psi _l}}},\,\prod\limits_{{l=1}}^{r} {{{({\mu _l}+{\eta _l})}^{{\psi _l}}}} - \prod\limits_{{l=1}}^{r} {{{({\eta _l})}^{{\psi _l}}}} } \right),$$

**Step 3** Estimate the criteria weights.

Assume that $w=\left\{ {{w_1},{w_2},\ldots,{w_n}} \right\}$ is the weight vector of the criteria set, satisfying $\sum\nolimits_{{j=1}}^{n} {{w_j}} =1$ and${w_j} \in \left[ {0,1} \right].$ To find the numeric values of criteria weights, we present the following three cases:

**Case I** Objective weight determination by the PFDM-based procedure.

With the use of Eq. (6), we compute the objective weight of the *j*^th^ criterion during the assessment of most suitable alternative.

10 $$w_{j}^{o}=\frac{{\frac{1}{{m - 1}}\sum\nolimits_{{i=1}}^{m} {\sum\nolimits_{{k=1}}^{m} {d\left( {{z_{ij}},\,{z_{kj}}} \right)} } }}{{\sum\nolimits_{{j=1}}^{n} {\left( {\frac{1}{{m - 1}}\sum\nolimits_{{i=1}}^{m} {\sum\nolimits_{{k=1}}^{m} {d\left( {{z_{ij}},\,{z_{kj}}} \right)} } } \right)} }},\quad j=1,2,\ldots,n.$$

Here, the term ‘$d\left( {{z_{ij}},\,{z_{kj}}} \right)$’ denotes the distance measure between PFNs ${z_{ij}}$ and ${z_{kj}}.$

**Case II** Subjective weight determination by the PF-RANCOM model.

Here, the classical RANCOM model has been extended within the picture fuzzy environment. It involves the following steps:

**Step 3.1** Using a PFWA operator, aggregate the picture fuzzy performance value of each criterion, provided by the DEs, and obtain an aggregated column matrix $G={\left( {{\xi _j}} \right)_{1 \times n}}.$ Using Eq. (3), compute the score value of each PFN of the aggregated column matrix $G={\left( {{\xi _j}} \right)_{1 \times n}}.$

**Step 3.2** Considering the descending score values, determine the rank of considered criteria.

**Step 3.3** By means of the pairwise comparison of criteria positions, generate the ranking comparison matrix (RCM). The comparison result is presented as ${\varphi _{kj}}$. Thus, the RCM can be represented as

11 $$\begin{gathered} \begin{array}{*{20}{c}} {\,\,\,\,\,\,\,\,\,\,\,\,\,\,\,\,\,\,\,\,\,\,\,\,\,\,\,\,\,\,\,\,\,\,\,\,\,{Q_1}}&{\,{Q_2}}&{\, \cdots }&{{Q_n}} \end{array} \hfill \\ {\varphi _{kj}}=\begin{array}{*{20}{c}} {{Q_1}} \\ {{Q_2}} \\ \vdots \\ {{Q_n}} \end{array}\left[ {\begin{array}{*{20}{c}} {{\theta _{11}}}&{{\theta _{11}}}& \cdots &{{\theta _{1n}}} \\ {{\theta _{21}}}&{{\theta _{22}}}& \cdots &{{\theta _{2n}}} \\ \vdots & \vdots & \ddots & \vdots \\ {{\theta _{n1}}}&{{\theta _{n2}}}& \cdots &{{\theta _{nn}}} \end{array}} \right], \hfill \\ \end{gathered}\quad\hbox{where}\quad {\varphi _{kj}}=\left\{ \begin{array}{ll} 1,& if\quad \tilde {\mathbb{S}}\left( {{Q_j}} \right)<\tilde {\mathbb{S}}\left( {{Q_k}} \right), \hfill \\ 0.5,& if\quad \tilde {\mathbb{S}}\left( {{Q_j}} \right)=\tilde {\mathbb{S}}\left( {{Q_k}} \right), \hfill \\ 0,& if\quad\tilde {\mathbb{S}}\left( {{Q_j}} \right)>\tilde {\mathbb{S}}\left( {{Q_k}} \right). \hfill \\ \end{array} \right.$$

**Step 3.4** Determine the summed criteria weight of the *j*^th^ criterion, where.

12 $${e_j}=\sum\limits_{{k=1}}^{n} {{\varphi _{kj}}} ,\quad j=1,2,\ldots,n.$$

**Step 3.5** Compute the subjective weight of the *j*^th^ criterion.

13 $$w_{j}^{s}=\frac{{{b_j}}}{{\sum\nolimits_{{j=1}}^{n} {{b_j}} }},\quad j=1,2,\ldots,n.$$

**Case III** By combining the objective and subjective weights of criteria, an integrated weighting formula is given as follows.

14 $${w_j}=\varsigma w_{j}^{o}+\left( {1 - \varsigma } \right)w_{j}^{s},$$

where $\varsigma$ denotes the precision factor of decision strategy.

**Step 4** Compute the optimal assessment rating (OAR) using Eq. (15).

15 $${E_0}=\left\{ \begin{array}{ll} \hbox{max} \,\tilde {\mathbb{S}}\left( {{z_{ij}}} \right),& j \in \hbox{Benefit criteria set}, \hfill \\ \hbox{min} \,\tilde {\mathbb{S}}\left( {{z_{ij}}} \right),& j\in \hbox{Cost criteria set}. \hfill \\ \end{array} \right.$$

**Step 5** Normalize the APF-DM.

If both the benefit and cost criteria are presented in the MCDM problem, then it is necessary to convert the APF-DM ${\mathbb{Z}}={\left( {{z_{ij}}} \right)_{m \times n}}$ into a normalized form ${\mathbb{N}}={\left( {{\varepsilon _{ij}}} \right)_{m \times n}}$, where

16 $${\varepsilon _{ij}}=\left( {{{\tilde {\mu }}_{ij}},\,{{\tilde {\eta }}_{ij}},\,{{\tilde {\nu }}_{ij}}} \right)=\left\{ \begin{array}{ll} {z_{ij}}=\left( {{\mu _{ij}},\,{\eta _{ij}},\,{\nu _{ij}}} \right),& {\text{for benefit criterion}}, \hfill \\ {z_{ij}}^{c}=\left( {{\nu _{ij}},\,{\eta _{ij}},\,{\mu _{ij}}} \right),&{\text{for cost criterion}}. \hfill \\ \end{array} \right.$$

**Step 6** Generate the weighted normalized APF-DM.

Apply the PFWA operator to the normalized APF-DM to construct the weighted normalized APF-DM $${\overset{\lower0.5em\hbox{$\smash{\scriptscriptstyle\frown}$}}{\mathbb{N}} _w}={\left( {{{\tilde {\varsigma }}_{ij}}} \right)_{m \times n}}$$, where

17 $${\overset{\lower0.5em\hbox{$\smash{\scriptscriptstyle\frown}$}}{\varsigma } _{ij}}=\left( {1 - {{\left( {1 - {{\overset{\lower0.5em\hbox{$\smash{\scriptscriptstyle\frown}$}}{\mu } }_{ij}}} \right)}^{{w_j}}},\,{{\left( {{{\overset{\lower0.5em\hbox{$\smash{\scriptscriptstyle\frown}$}}{\eta } }_{ij}}} \right)}^{{w_j}}},{{\left( {{{\overset{\lower0.5em\hbox{$\smash{\scriptscriptstyle\frown}$}}{\mu } }_{ij}}+{{\overset{\lower0.5em\hbox{$\smash{\scriptscriptstyle\frown}$}}{\eta } }_{ij}}} \right)}^{{w_j}}} - {{\left( {{{\overset{\lower0.5em\hbox{$\smash{\scriptscriptstyle\frown}$}}{\eta } }_{ij}}} \right)}^{{w_j}}}} \right)=\left( {{{\bar {\mu }}_{ij}},{{\bar {\eta }}_{ij}},\,{{\bar {\nu }}_{ij}}} \right).$$

**Step 7** Compute the score value of each element of the weighted normalized APF-DM $${\overset{\lower0.5em\hbox{$\smash{\scriptscriptstyle\frown}$}}{\mathbb{N}} _w}={\left( {{{\tilde {\varsigma }}_{ij}}} \right)_{m \times n}}$$ using Eq. (3).

**Step 8** Based on Eq. (18), determine the overall assessment values of alternatives.

18 $${Y_i}=\sum\limits_{{j=1}}^{n} {\varphi \left( {{{\tilde {\varsigma }}_{ij}}} \right)} ,$$

where $\varphi \left( {{{\tilde {\varsigma }}_{ij}}} \right)$ denotes the score value of the *i*^th^ element of the weighted normalized APF-DM concerning the *j*^th^ criterion.

**Step 9** Calculate the utility degrees of alternatives through Eq. (19).

19 $${V_i}=\frac{{{Y_i}}}{{{E_0}}},\quad i=1,2,\ldots,m.$$

According to the decreasing values of utility degrees, determine the ranking order of alternatives. The alternative with the highest utility degree is considered as the most suitable choice.

## Case study: assessment of DT project risks

In this section, the PF-DM-RANCOM-ARAS model is applied to the case study of a medium-sized manufacturing company located in Chennai, India. The company works in diverse sectors, such as automotive, renewable energy and white goods. This manufacturing company produces electromechanical machinery where temperature sensors and magnetic switches are formed. Since the electronics sector has struggled with digital adoption, professionals have taken a positive approach in the direction of the scope of the present study. Though the digital transitions are challenged by low technological maturity levels within their company, the executives are keen to study and implement innovative technologies on the shop floor. As the risks of adopting DT projects consist of several questions, the present PF-DM-RANCOM-ARAS can be assumed as an appropriate model to assess and rank DT projects from the risk viewpoint.

To identify and assess the risks of DT projects, we conducted a survey by means of current literature and discussions with experts. Firstly, a comprehensive list including 15 risks was determined from recent works. Next, these risks were used to create a questionnaire layout and then communicated to 10 different experts working in the field of electronics and digital economy transformation and having more than ten years of expertise in their respective disciplines. To identify the experts, we explored the published articles related to DT on ResearchGate and Google Scholar. We sent email invitations to some experts for participating in this survey, however, only 4 of them replied to our requests. These four DEs (*b*_1_, *b*_2_, *b*_3_, *b*_4_) are highly experienced in the project assessment, manufacturing technology and digital facilities and their disciplines. Based on their experience, expertise and knowledge, the researchers provided assessment ratings of each DE in the form of LVs using the Likert scale presented in Table 2 to assess the risks of DT projects. In the next stage, we sent questionnaire forms to obtain their opinions. In the main questionnaire form, we identified 15 risks of DT projects. Further, after scrutinizing all questionnaire forms, we considered 11 risks as criteria for the MCDM problem, given in Table 1; Fig. 1. According to this team, three DT project options were considered, which are depicted in Sect. 3 (see Fig. 1). This section offers the outcomes of the model when it is applied to a case study. Accordingly, this work describes the novel PF-RANCOM-ARAS method for assessing and prioritizing DT project risks and computing the results using MATLAB 2015a software^51^.

**Steps 1–2** Table 2 (adopted from Mishra et al.^17^) portrays the linguistic values and their corresponding PFNs. Table 3 presents the linguistic matrix given by the experts to prioritize DT projects over different risks.

**Table 3:** Linguistic matrix for DT project risks assessment.

Risks	*P*_1_				*P*_2_				*P*_3_			
*Q*_1_	AH	VH	VH	AH	A	VVH	VVH	VH	H	VVH	VH	VVH
*Q*_2_	VH	H	H	VH	AH	L	VH	A	AH	H	VVH	H
*Q*_3_	VVH	VH	VH	VVH	H	VVH	AH	VVH	H	VH	VH	VH
*Q*_4_	A	QL	QL	A	VH	H	VH	VVH	VVH	VH	A	VVH
*Q*_5_	QL	A	L	H	VH	AH	VH	H	A	H	AH	VH
*Q*_6_	VVH	AH	VH	VH	VH	H	VH	VH	A	AH	AH	H
*Q*_7_	VH	VVH	H	VH	A	H	VH	AH	VH	VVH	VH	AH
*Q*_8_	A	AH	H	H	VVH	VVH	VH	VVH	VVH	A	VH	VH
*Q*_9_	H	VVH	AH	VVH	H	A	AH	VH	AH	VVH	H	H
*Q*_10_	AH	VL	VL	A	A	VL	A	A	VL	AH	L	QL
*Q*_11_	VL	AH	QL	A	AH	A	H	AH	A	L	AH	L

Based on the knowledge and expertise of the invited DEs, we first provide the linguistic assessment rating of each DE using Table 2. Further, the weights of DEs are computed using Eq. (8) and shown in Table 4. Afterwards, the APF-DM is constructed using Tables 2, 3 and 4 and Eq. (9) and given in Table 5.

**Table 4:** DEs’ weights for the assessment of DT projects from risks perspective.

DEs	LRs	PFNs	Weights
*b*_1_	VH	(0.80, 0.05, 0.10)	0.245
*b*_2_	VVH	(0.90, 0.05, 0.05)	0.302
*b*_3_	H	(0.75, 0.10, 0.10)	0.208
*b*_4_	VH	(0.80, 0.05, 0.10)	0.245

**Table 5:** APF-DM for the DT project risks assessment.

	*P*_1_	*P*_2_	*P*_3_
*Q*_1_	(0.719, 0.070, 0.140)	(0.817, 0.059, 0.108)	(0.855, 0.059, 0.070)
*Q*_2_	(0.776, 0.071, 0.103)	(0.567, 0.070, 0.302)	(0.793, 0.087, 0.087)
*Q*_3_	(0.858, 0.050, 0.073)	(0.833, 0.068, 0.080)	(0.831, 0.050, 0.086)
*Q*_4_	(0.365, 0.070, 0.545)	(0.819, 0.062, 0.087)	(0.828, 0.058, 0.100)
*Q*_5_	(0.492, 0.073, 0.393)	(0.740, 0.073, 0.125)	(0.691, 0.084, 0.181)
*Q*_6_	(0.792, 0.062, 0.106)	(0.786, 0.062, 0.102)	(0.623, 0.100, 0.208)
*Q*_7_	(0.830, 0.058, 0.083)	(0.683, 0.087, 0.174)	(0.884, 0.050, 0.059)
*Q*_8_	(0.659, 0.100, 0.183)	(0.884, 0.050, 0.059)	(0.687, 0.073, 0.183)
*Q*_9_	(0.833, 0.068, 0.080)	(0.678, 0.084, 0.183)	(0.787, 0.081, 0.098)
*Q*_10_	(0.38, 0.075, 0.488)	(0.473, 0.081, 0.407)	(0.355, 0.062, 0.503)
*Q*_11_	(0.405, 0.073, 0.458)	(0.612, 0.100, 0.222)	(0.426, 0.068, 0.451)

**Step 3**: Considering the PF-distance measure-based formula, we determine the objective weight of risks in order to assess the DT projects by using Eqs. (5) and (10), given as follows: $w_{j}^{o}$ = (0.0513, 0.1077, 0.0136, 0.2194, 0.1239, 0.0735, 0.0831, 0.094, 0.0643, 0.0575, 0.1116). To find the subjective weight of each criterion, the PF-RANCOM tool is used. In this regard, each DE presents their opinion regarding the performance of each considered criterion, which is further converted into an aggregated PFN using a PFWA operator. Next, the score value of each aggregated PFN is determined using Eq. (3) and ranked accordingly. The last two columns of Table 6 present the score value and rank of each criterion, respectively, to assess the risk criteria. On the basis of comparisons made by the DEs, the RCM is created through Eq. (11). In accordance with the RCM, the summed criteria weights are estimated using Eq. (12). Lastly, we obtain a subjective weight of each criterion by means of Eq. (13) and given as $w_{j}^{s}$ = (0.0579, 0.1405, 0.0744, 0.0909, 0.1074, 0.1240, 0.1736, 0.1570, 0.0248, 0.0083, 0.0413). Table 7 presents the required computational results of the PF-RANCOM model.

**Table 6:** Aggregated values and ranks of the risks obtained by DEs.

Risks	b_1_	b_2_	b_3_	b_4_	APF-DM	Crisp values	Rank
*Q*_1_	A	H	VH	AH	(0.683, 0.087, 0.174)	0.807	8
*Q*_2_	VVH	H	AH	VH	(0.791, 0.071, 0.100)	0.873	3
*Q*_3_	H	A	VH	H	(0.706, 0.087, 0.162)	0.819	7
*Q*_4_	A	H	VH	VH	(0.732, 0.073, 0.147)	0.837	6
*Q*_5_	A	VH	H	VH	(0.738, 0.068, 0.145)	0.841	5
*Q*_6_	VH	H	H	H	(0.763, 0.084, 0.102)	0.859	4
*Q*_7_	VH	VVH	VH	VH	(0.838, 0.050, 0.083)	0.902	1
*Q*_8_	VH	VH	H	VH	(0.790, 0.058, 0.101)	0.877	2
*Q*_9_	VL	A	L	H	(0.477, 0.073, 0.407)	0.666	10
*Q*_10_	AH	QL	L	A	(0.415, 0.070, 0.456)	0.629	11
*Q*_11_	L	AH	A	AH	(0.519, 0.084, 0.319)	0.705	9

**Table 7:** Estimation of RCM and summed weight of each criterion for DT project risks assessment.

Risks	RCM											${e_j}$	$w_{j}^{s}$
	Q_1_	Q_2_	Q_3_	Q_4_	Q_5_	Q_6_	Q_7_	Q_8_	Q_9_	Q_10_	Q_11_		
*Q*_1_	0.5	0	0	0	0	0	0	0	1	1	1	3.5	0.0579
*Q*_2_	1	0.5	1	1	1	1	0	0	1	1	1	8.5	0.1405
*Q*_3_	1	0	0.5	0	0	0	0	0	1	1	1	4.5	0.0744
*Q*_4_	1	0	1	0.5	0	0	0	0	1	1	1	5.5	0.0909
*Q*_5_	1	0	1	1	0.5	0	0	1	1	1	1	7.5	0.1074
*Q*_6_	1	0	1	1	1	0.5	0	0	1	1	1	7.5	0.1240
*Q*_7_	1	1	1	1	1	1	0.5	1	1	1	1	10.5	0.1736
*Q*_8_	1	1	1	1	1	1	0	0.5	1	1	1	9.5	0.1570
*Q*_9_	0	0	0	0	0	0	0	0	0.5	1	0	1.5	0.0248
*Q*_10_	0	0	0	1	0	0	0	0	0	0.5	0	1.5	0.0083
*Q*_11_	0	0	0	0	0	0	0	0	1	1	0.5	2.5	0.0413

Combining the objective and subjective weights of criteria by means of Eq. (14), the combined weight of each criterion is computed as *w*_*j*_ = (0.0546, 0.1241, 0.044, 0.1552, 0.1156, 0.0988, 0.1284, 0.1255, 0.0446, 0.0329, 0.0764). Here, $\varsigma=0.5.$

**Fig. 3:**
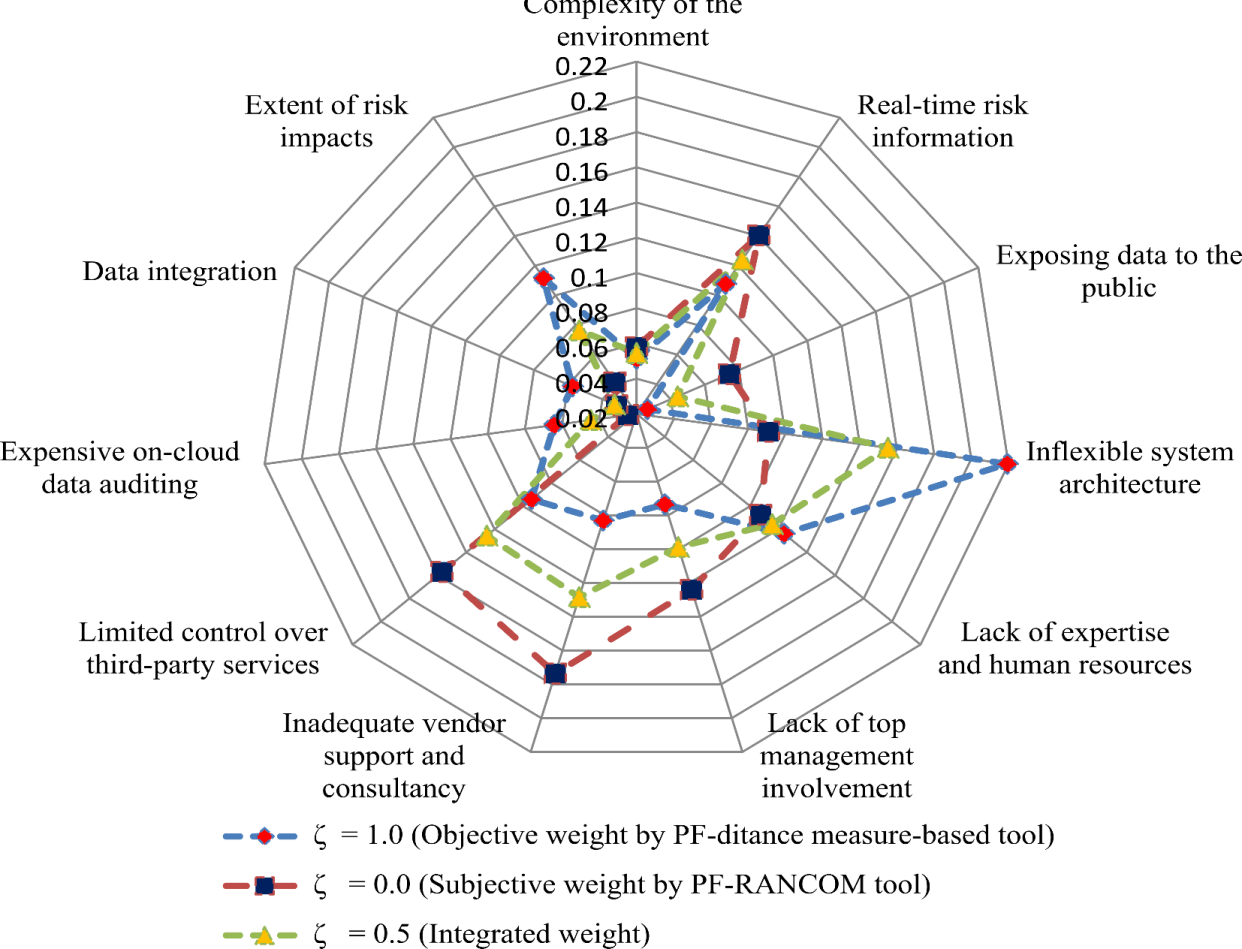
Assessment of weights of different DT project risks.

Figure 3 shows the different weights of considered risk factors for evaluating the DT project alternatives from the risks perspective. Inflexible system architecture (*Q*_4_) (0.1552) is the most significant risk for assessing the DT project alternatives. Inadequate vendor support and consultancy (*Q*_7_) (0.1284) is the second most significant risk factor for DT project risks assessment. Limited control over third-party services (*Q*_8_) (0.1255) is the third, Real-time risk information (*Q*_2_) (0.1241) is the fourth, and Lack of expertise and human resources (*Q*_5_) (0.1156) is the fifth most important risk factor for DT project risks assessment, while the remaining factors are crucial risk factors for assessing the DT project alternatives.

**Step 4** The OAR of options to use the DT project risks for DT project risks assessment is determined using Eq. (15) as *E*_0_ = {(0.855, 0.059, 0.07), (0.793, 0.087, 0.087), (0.858, 0.05, 0.073), (0.828, 0.058, 0.1), (0.74, 0.073, 0.125), (0.792, 0.062, 0.106), (0.884, 0.05, 0.059), (0.884, 0.05, 0.059), (0.833, 0.068, 0.08), (0.473, 0.081, 0.407), (0.612, 0.1, 0.222)}.

**Steps 5–7** Since all the risk factors for DT project risks assessment are benefit-type, Eq. (16) is not used to make the normalized APF-DM. A weighted normalized APF-DM is created using Eq. (17), as shown in Table 8, and the score values of each PFN of the weighted normalized APF-DM are further computed.

**Table 8:** Weighted normalized APF-DM for DT project risks assessment.

Risks	E_0_	*P*_1_	*P*_2_	*P*_3_
*Q*_1_	(0.099, 0.858, 0.037)	(0.066, 0.866, 0.053)	(0.088, 0.859, 0.050)	(0.099, 0.859, 0.037)
*Q*_2_	(0.173, 0.744, 0.065)	(0.165, 0.727, 0.083)	(0.096, 0.725, 0.162)	(0.174, 0.744, 0.065)
*Q*_3_	(0.071, 0.893, 0.031)	(0.071, 0.893, 0.031)	(0.066, 0.903, 0.027)	(0.065, 0.893, 0.034)
*Q*_4_	(0.256, 0.620, 0.114)	(0.073, 0.640, 0.282)	(0.250, 0.626, 0.099)	(0.256, 0.619, 0.114)
*Q*_5_	(0.146, 0.736, 0.091)	(0.076, 0.736, 0.179)	(0.146, 0.736, 0.091)	(0.129, 0.748, 0.108)
*Q*_6_	(0.137, 0.771, 0.075)	(0.137, 0.770, 0.076)	(0.135, 0.770, 0.074)	(0.087, 0.806, 0.090)
*Q*_7_	(0.227, 0.699, 0.068)	(0.191, 0.712, 0.080)	(0.128, 0.747, 0.105)	(0.227, 0.699, 0.068)
*Q*_8_	(0.226, 0.700, 0.068)	(0.120, 0.760, 0.100)	(0.227, 0.700, 0.068)	(0.129, 0.732, 0.118)
*Q*_9_	(0.083, 0.878, 0.034)	(0.083, 0.878, 0.034)	(0.054, 0.887, 0.051)	(0.072, 0.885, 0.035)
*Q*_10_	(0.024, 0.909, 0.065)	(0.018, 0.906, 0.073)	(0.024, 0.909, 0.065)	(0.017, 0.900, 0.079)
*Q*_11_	(0.076, 0.825, 0.085)	(0.042, 0.804, 0.145)	(0.076, 0.825, 0.085)	(0.045, 0.799, 0.147)

**Steps 8–9** Next, using Eq. (18), the overall assessment values of DT project alternatives are calculated and shown in Table 9. By Eq. (19), the utility degree of each alternative is found as *Y*_1_ = 0.9339, *Y*_2_ = 0.9697 and *Y*_3_ = 0.9694. Based on the UDs, the prioritization of DT project alternatives in terms of risks assessment is ${P_2} \succ {P_3} \succ {P_1},$ and, thus, the “Augmented reality-based warehouse management (*P*_2_)” is the optimal project from the risks perspective within the picture fuzzy environment.

**Table 9:** OAR of project options for DT project risks assessment.

Risks	E_0_	*P*_1_	*P*_2_	*P*_3_
*Q*_1_	0.401	0.382	0.393	0.401
*Q*_2_	0.455	0.452	0.403	0.455
*Q*_3_	0.383	0.383	0.378	0.379
*Q*_4_	0.508	0.384	0.508	0.507
*Q*_5_	0.440	0.387	0.440	0.424
*Q*_6_	0.430	0.430	0.430	0.397
*Q*_7_	0.486	0.466	0.425	0.487
*Q*_8_	0.486	0.420	0.486	0.426
*Q*_9_	0.391	0.390	0.372	0.384
*Q*_10_	0.351	0.347	0.351	0.346
*Q*_11_	0.389	0.365	0.389	0.366
OAI	4.718	4.406	4.575	4.574
Utility degree	-	0.9339	0.9697	0.9694
Ranking	-	3	1	2

## Sensitivity and comparative discussion

In the current section, we will first discuss the sensitivity analysis with respect to varying values of the decision strategy parameter. Then, we will compare the proposed and existing PF-information based methods. Finally, we will present the policy implications of the proposed work.

### Sensitivity investigation

**Fig. 4:**
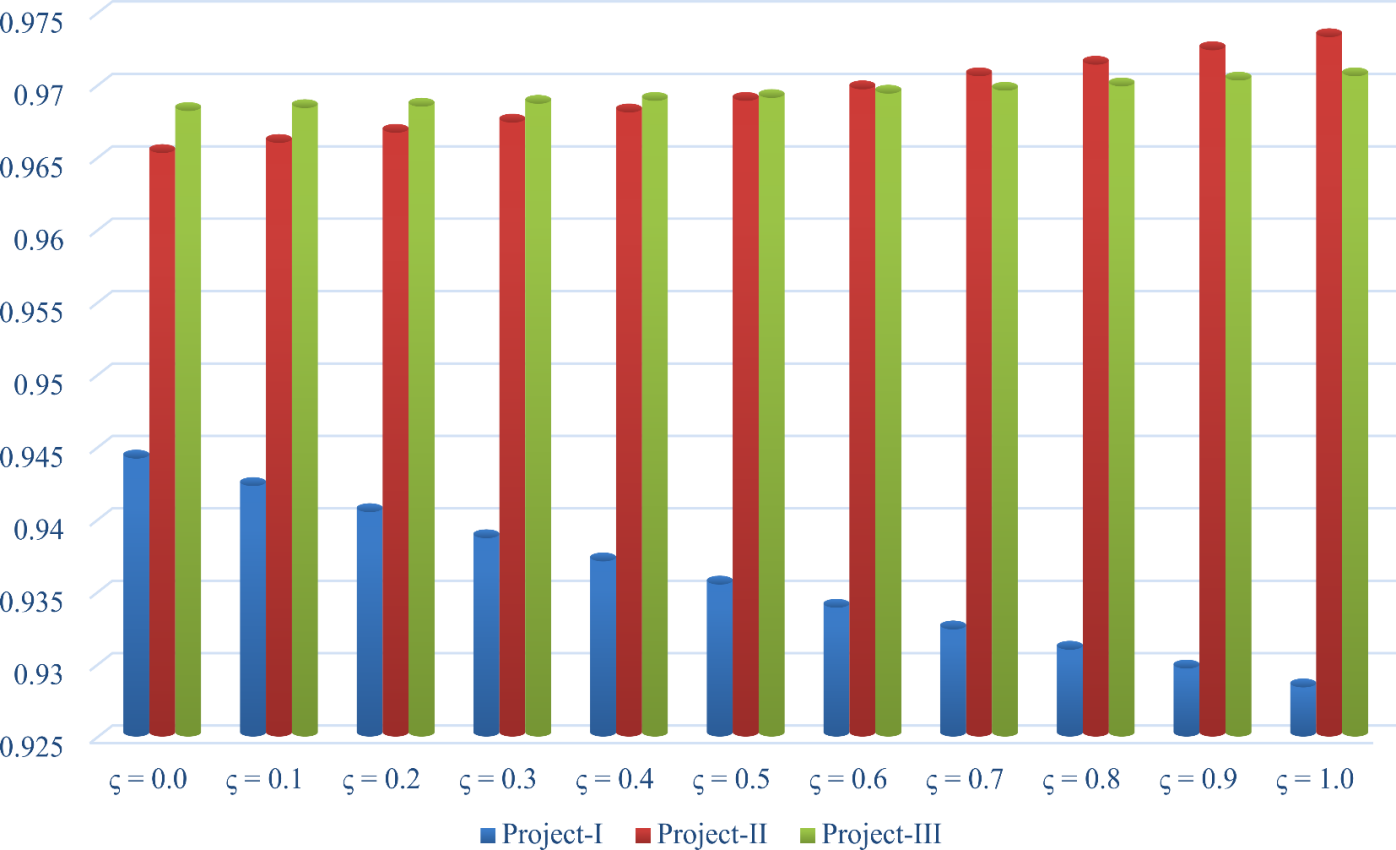
Sensitivity of the weight parameter (*ς*) for DT project risks assessment.

This subsection discusses the sensitivity analysis to check the impact of varying criteria weights on the final results. Table 10; Fig. 4 present the required results of the sensitivity analysis. For this purpose, we consider the following cases:

***Case-I*** In this case, we consider the PFDM-based tool for finding the objective weight of criteria, i.e., = 1.0 in Eq. (14) during the assessment of DT projects from the risks perspective. Using this model, the overall assessment degree of each option is computed and given as *Y*_1_ = 0.9284, *Y*_2_ = 0.9733 and *Y*_3_ = 0.9706. On the basis of the decreasing values of overall assessment degrees, the prioritization order of DT projects is *P*_2_$\succ$*P*_3_$\succ$*P*_1_. Hence, the “Augmented reality-based warehouse management (*P*_2_)” is the most optimal project.

***Case-II*** In this case, we consider the PF-RANCOM model to compute the subjective weight of criteria, i.e., = 0.0 in Eq. (14) during the assessment of DT projects from the risks perspective. Using this model, the overall assessment degree of each option is computed and given as *Y*_1_ = 0.9442, *Y*_2_ = 0.9653 and *Y*_3_ = 0.9682. Based on the decreasing values of overall assessment degrees, the prioritization order of DT projects is *P*_3_$\succ$*P*_2_$\succ$*P*_1_. Hence, the “IoT-based preventive maintenance (P3)” is the most optimal project.

***Case-III*** In this case, we consider the integrated PF-DM-RANCOM model to compute the final weight of criteria, i.e., = 0.5 in Eq. (14) during the assessment of DT projects from the risks perspective. Using this model, the overall assessment degree of each option is computed and given as *Y*_1_ = 0.9339, *Y*_2_ = 0.9697 and *Y*_3_ = 0.9694. By means of the decreasing values of overall assessment degrees, the prioritization order of DT projects is *P*_2_$\succ$*P*_3_$\succ$*P*_1_. Hence, the “Augmented reality-based warehouse management (*P*_2_)” is the most optimal project. On the basis of the above investigations (see Table 10; Fig. 4), it can be observed that by changing the values of the weighting parameter, the performance of the developed ranking framework improves.

**Table 10:** The OADs of project options over the weighting parameter (*ς*).

Options	ς = 0.0	ς = 0.1	ς = 0.2	ς = 0.3	ς = 0.4	ς = 0.5	ς = 0.6	ς = 0.7	ς = 0.8	ς = 0.9	ς = 1.0
*P*_1_	0.9442	0.9423	0.9405	0.9387	0.9371	0.9355	0.9339	0.9324	0.9310	0.9297	0.9284
*P*_2_	0.9653	0.9660	0.9667	0.9674	0.9681	0.9689	0.9697	0.9706	0.9714	0.9724	0.9733
*P*_3_	0.9682	0.9684	0.9685	0.9687	0.9689	0.9691	0.9694	0.9696	0.9699	0.9703	0.9706

### Comparative study

In this section, the comparison is made between the proposed and existing PF-information based approaches. To this aim, we chose some of the existing approaches described by Wang et al.^52^, Lu et al.^53^, Hezam et al.^54^ and Svadlenka et al.^55^, and applied them to the DT project risks assessment problem.

#### PF-VIKOR tool

In this part of study, Wang et al.’s PF-VIKOR method^52^ is applied to the aforesaid case study. Using this method, the ideal and anti-ideal solutions are computed as ${\phi ^+}$ = {(0.855, 0.059, 0.07), (0.793, 0.087, 0.087), (0.858, 0.05, 0.073), (0.828, 0.058, 0.1), (0.740, 0.073, 0.125), (0.792, 0.062, 0.106), (0.884, 0.05, 0.059), (0.884, 0.05, 0.059), (0.833, 0.068, 0.08), (0.483, 0.081, 0.404), (0.612, 0.1, 0.222)} and ${\phi ^ - }$={(0.719, 0.07, 0.14), (0.567, 0.070, 0.302), (0.833, 0.068, 0.08), (0.365, 0.07, 0.545), (0.492, 0.073, 0.393), (0.623, 0.1, 0.208), (0.683, 0.087, 0.174), (0.659, 0.1, 0.183), (0.678, 0.084, 0.183), (0.356, 0.062, 0.502), (0.405, 0.073, 0.458)}. Further, the group utility of each alternative is calculated as 1.652, 0.172 and 0.775, respectively. Next, the individual regret of each option is determined as 0.573, 0.224 and 0.612, respectively. Considering the group utility and individual regret of each option, the compromise scores of alternatives are computed as 0.95, 0.0 and 0.704, respectively, and finally, the ranking order of alternatives is *P*_2_$\succ$*P*_3_$\succ$*P*_1_.

#### PF-COPRAS tool

Here, Lu et al.’s PF-COPRAS method^53^ is applied to the aforesaid case study. Using this method, the relative degree of each option is obtained as 0.771, 0.841 and 0.837 and further the utility degree of each option is computed as 91.78, 100.0 and 99.58. Thus, the prioritization order of project options for DT project risks assessment is *P*_2_$\succ$*P*_3_$\succ$*P*_1_. It implies that the “Augmented reality-based warehouse management (*P*_2_)” option is the optimal project from the risks perspective within the picture fuzzy environment.

#### PF-WASPAS tool

In accordance with the Hezam et al.’s PF-WASPAS method^54^, the additive relative importance of each project alternative is computed as (0.672, 0.07, 0.212) (0.754, 0.069, 0.137) and (0.759, 0.069, 0.140), and the related score values are 0.797, 0.849 and 0.85, respectively. Next, the multiplicative relative importance of each project alternative is determined as (0.599, 0.070, 0.288), (0.726, 0.069, 0.16) and (0.713, 0.069, 0.176), and the related score values are 0.747, 0.832 and 0.823, respectively. Further, the total relative importance of each project option is determined as 0.7716, 0.8408 and 0.836, respectively. Thus, the prioritization order of project options for DT project risks assessment is *P*_2_$\succ$*P*_3_$\succ$*P*_1_. Hence, the “Augmented reality-based warehouse management (*P*_2_)” is the optimal choice from the risks perspective within the picture fuzzy environment.

#### PF-CoCoSo model

**Fig. 5:**
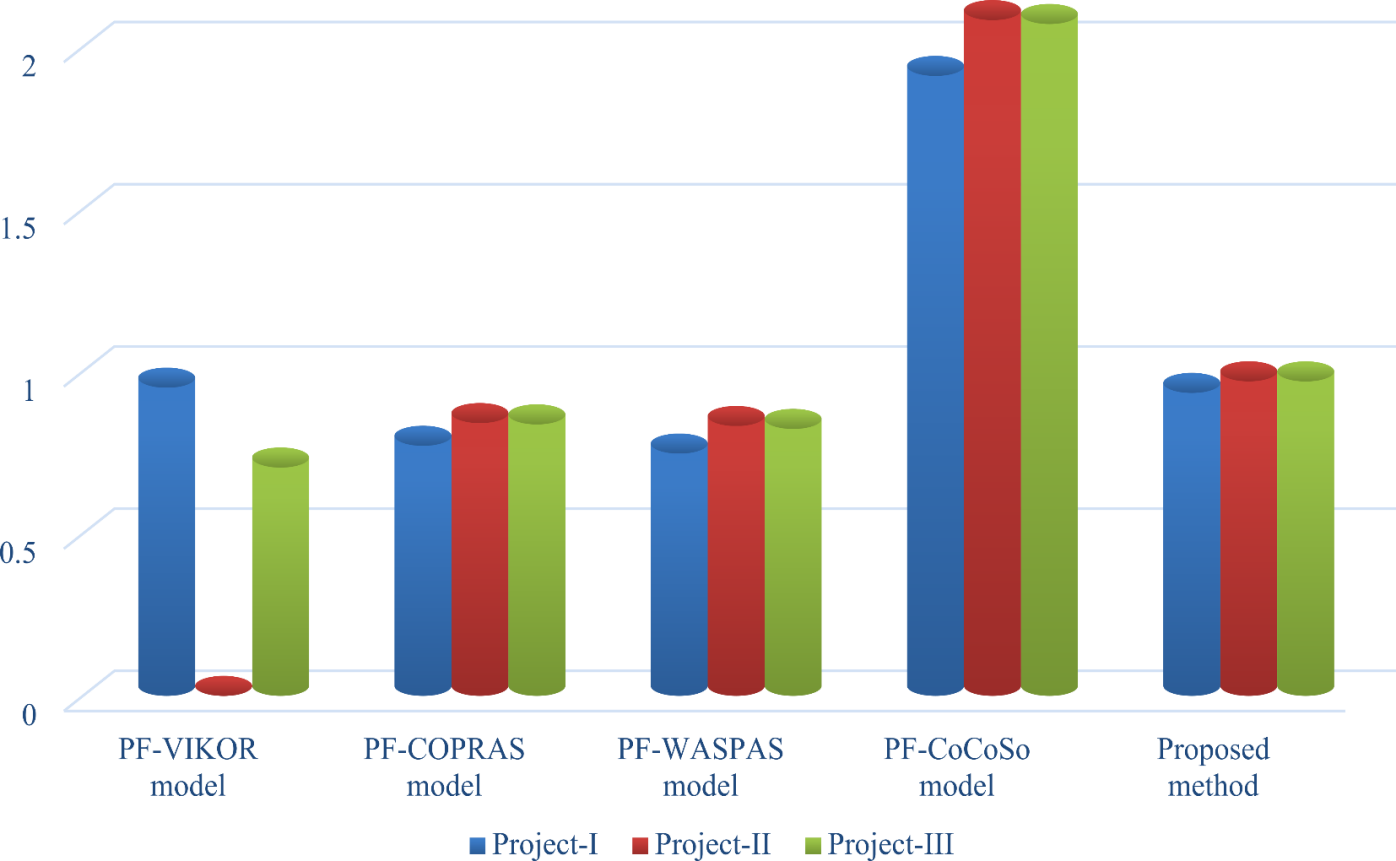
Results of the proposed and existing PF-information based methods.

The PF-CoCoSo method^55^ also computes the additive and multiplicative importance rating of alternatives like the PF-WASPAS method. Therefore, these values are similar to Hezam et al.^53^. Next, the relative compromise degrees of alternatives are computed as $r_{1}^{{\left( 1 \right)}}$ = 0.315, $r_{2}^{{\left( 1 \right)}}$ = 0.343, $r_{3}^{{\left( 1 \right)}}$ = 0.341, $r_{1}^{{\left( 2 \right)}}$ = 2.0, $r_{2}^{{\left( 2 \right)}}$ = 2.181, $r_{3}^{{\left( 2 \right)}}$ = 2.168, $r_{1}^{{\left( 3 \right)}}$ = 0.918, $r_{2}^{{\left( 3 \right)}}$ = 1.0 and $r_{3}^{{\left( 3 \right)}}$ = 0.994. Further, the overall compromise rating of each alternative is computed as 1.911, 2.083 and 2.071, respectively. Thus, the “Augmented reality-based warehouse management (*P*_2_)” is the optimal choice and the ranking order of project options is *P*_2_$\succ$*P*_3_$\succ$*P*_1_. Figure 5 shows the results of the proposed and existing methods.

From the above discussions, it can be noticed that project-II (Augmented reality-based warehouse management (*P*_2_)) is the best option.

In what follows, we will present the theoretical comparison between the proposed and existing approaches:

- The proposed method computes the DEs’ weights during the assessment of alternatives, while the former methods by Wang et al.^52^ and Lu et al.^53^ consider the assumed DEs’ weights, whereas Hezam et al.^54^ assign an equal weight to each DE, which may cause a loss of information. Hence, the precision and consistency of outcomes by the proposed method are more applicable.
- In Lu et al.^53^, the CRITIC is utilized to find the objective weight of criteria for DT project risks assessment. In Wang et al.^52^, an entropy-model is applied to estimate the objective weight of criteria for DT project risks assessment. In Svadlenka et al.^55^, the criteria weight vector is estimated using a PF-score function-based procedure. In Hezam et al.^54^, a PF-similarity degree-based tool is used to find the objective weight of criteria for DT project risks assessment. In the developed model, the PF-DM-RANCOM tool is used to find the objective weight by a PFDM-based model and the subjective weight by a PF-RANCOM model, which proves that the developed model is more reasonable, proficient, and applicable.
- For PF-VIKOR, it is essential to determine the distance on each option and reference points, which is a complex process that reduces the accuracy of outcomes, whereas PF-COPRAS can determine complex associations from PFDM using a PFWA operator. In PF-WASPAS and PF-CoCoSo methods, the utility degree of options is determined on the basis of PF-weighted averaging and geometric operators. PF-TOPSIS and PF-COPRAS models have two noteworthy drawbacks: (a) preferences of alternatives may vary over possible transformations of initial ratings of criteria in measurement-theoretic measure of the terms; and (b) the ranking order of options may change if new option is added to defined set of options or an earlier one is removed from it or swapped. In this study, we propose a hybrid PF-RANCOM-ARAS framework that possesses easy and instinctive procedures that produce acceptable, reasonable, and relatively accurate results in assessing and choosing suitable options with their performance ratings. The hybrid PF-RANCOM-ARAS framework offers a concept of UDs to rank different options.

### Discussion

The findings of the developed PF-DM-RANCOM-ARAS approach illustrate that the “Augmented reality-based warehouse management (*P*_2_)” is the most insecure project from the risks perspective. Project-II provides insightful variations to warehouse procedures as well as resources management. Alternatively, the “IoT-based preventive maintenance (*P*_3_)” project is considered to be ‘‘Fair” in accordance with risk magnitude. The key concern in project-III is the misalliance between firms’ present processes and software’s competences, which results in an extensive chaos. Since the enterprise is presently implementing the IoT-based predictive maintenance project, various procedures are standardized and carried out with the IoT-based predictive maintenance structure.

Further, the PF-DM-RANCOM-ARAS model ranks the alternatives by means of the objective and subjective weights of risk factors. Based on the developed PFDM and PF-RANCOM tools, we introduced an innovative weight-evaluating model to find significance degrees of DT project risks in India, taken from the literature survey and the DEs views. Then, the ARAS model was developed on the PFSs setting. In the ARAS model, a utility degree was implemented to prioritize the alternatives quantitatively for assessing the DT project options and risk factors. In this paper, data was collected physically, which needs enhancement. Online platforms will be considered to mechanize the developed approach in the future.

**Fig. 6:**
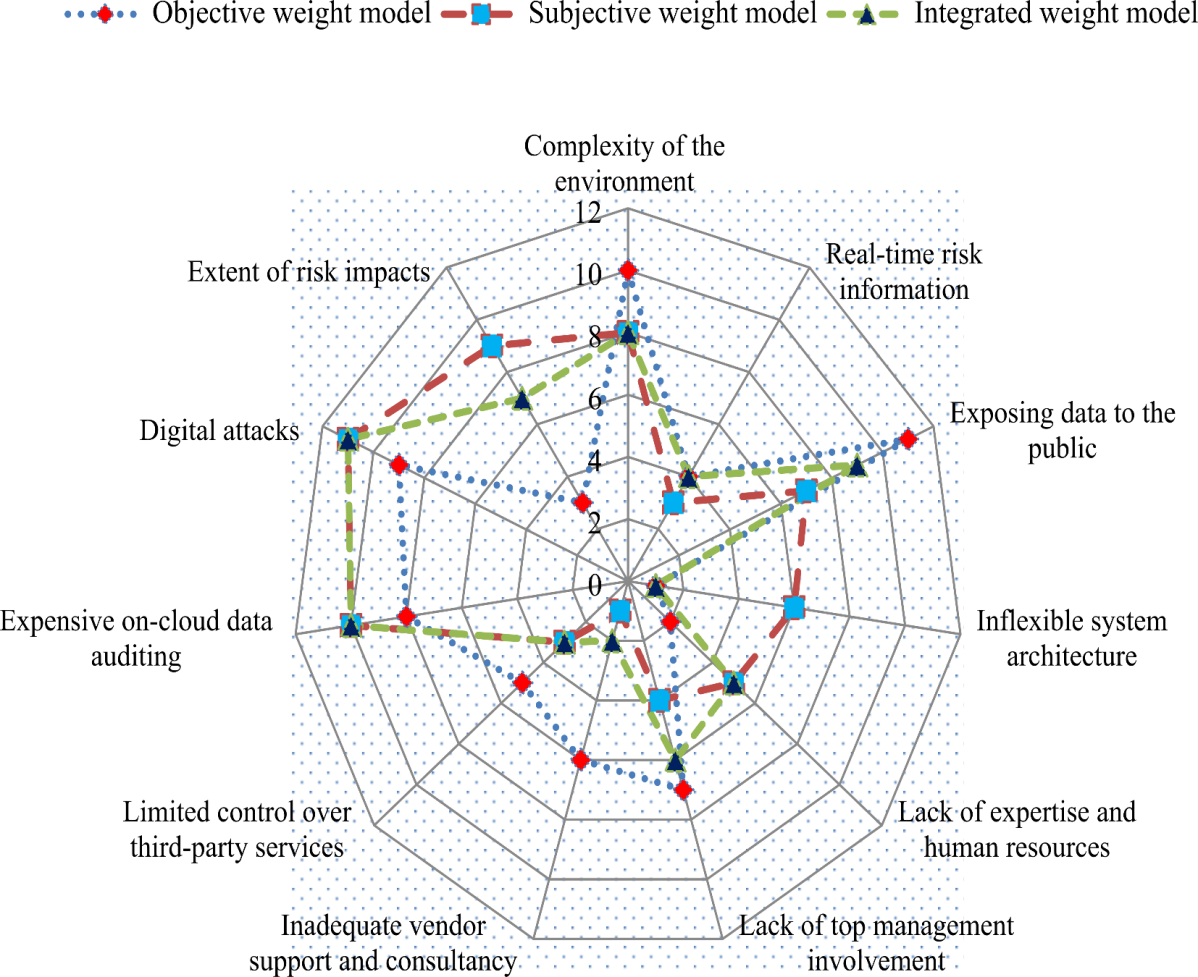
Represents the ranking of risk factors in assessing the DT project risks.

The significance degree or weight outcome shows that Inflexible system architecture (0.1552) is the most significant risk factor, as seen in Table 11, while the prioritization of risk factors is given in Fig. 6. This outcome is consistent with Gölcük’s model^27^ that discusses the DT project risks assessment. Inflexible system architecture is followed by Inadequate vendor support and consultancy (0.1284), Limited control over third-party services (0.1255), Real-time risk information (0.1241), Lack of expertise and human resources (0.1156), Lack of top management involvement (0.0519), Complying with law and regulations (0.0512), Training and development (0.0988), Extent of risk impacts (0.0764), Complexity of the environment (0.0546), Expensive on-cloud data auditing (0.0446), Exposing data to the public (0.044), and Digital attacks (0.0329).

**Table 11:** Significance ratings and rankings of DT project risks.

Risks	Objective weight	Ranking	Subjective weight	Ranking	Integrated weight	Ranking
Complexity	0.0513	10	0.0579	8	0.0546	8
Real-time risk information	0.1077	4	0.1405	3	0.1241	4
Exposing data to the public	0.0136	11	0.0744	7	0.044	9
Inflexible system architecture	0.2194	1	0.0909	6	0.1552	1
Budget constraints	0.1239	2	0.1074	5	0.1156	5
Lack of digital mindset	0.0735	7	0.124	4	0.0988	6
Ineffective vendor management	0.0831	6	0.1736	1	0.1284	2
Limited control over third-party services	0.094	5	0.157	3	0.1255	3
Lack of participation of top management	0.0643	8	0.0248	10	0.0446	10
Digital attacks	0.0575	9	0.0083	11	0.0329	11
Extent of risk impacts	0.1116	3	0.0413	9	0.0764	7

## Conclusions

Digital transformation (DT) is defined as the way by which firms implement technologies through their business operations to drive vital transformation. It can considerably enhance an organization’s competence by mechanizing manual procedures, reducing errors and enhancing production. These transformations present long-term determinations on how an organization constantly advances and changes. In recent years, a rapid transformation has been witnessed in the business domain with various firms implementing innovative technologies such as artificial intelligence, machine learning, BD and the IoT to achieve insights into customer behavior, streamline processes and advance decision-making. This paper developed a new hybrid PF-RANCOM-ARAS methodology to assess the DT projects from the risks viewpoint. The proposed methodology computed the DEs weights through a picture fuzzy score function-based process. The significance of considered risks was evaluated based on a combined weighting model consisting of a picture fuzzy distance measure-based tool for objective weights and the RANCOM approach for subjective weights of risks. Further, a novel picture fuzzy ARAS methodology was proposed to assess and prioritize the DT project alternatives by means of the risks perspective. To demonstrate the applicability of the model, a case study of a DT project risks assessment problem of a medium sized manufacturing company in Chennai (India) was examined. Sensitivity assessment over diverse values of the decision strategy parameter was discussed to prove the robustness of the obtained results. The proposed methodology was compared with diverse extant models, which confirmed its feasibility and strength. The obtained results demonstrated the efficacy and solidity of the model by considering the different risk factor weights, which were consistent with the existing methods. The stakeholders can use this model to understand and assess DT project risks.

In the case study, the Oracle fusion cloud enterprise resource planning software, Augmented reality-based warehouse management, and IoT-based preventive maintenance were assessed. The outcomes showed that the Augmented reality-based warehouse management project included a major risk. Alternatively, IoT-based predictive maintenance posed a fair risk. Lastly, the Oracle fusion cloud enterprise resource planning software project included a minor risk. Based on the findings, the prioritization of DT projects was estimated as Augmented reality-based warehouse management $\succ$ IoT-based preventive maintenance $\succ$ Oracle fusion cloud enterprise resource planning software.

This study has some limitations: (i) the presented PF-DM-RANCOM-ARAS approach does not reflect the interrelationships between risks, (ii) the considered alternatives, risk factors and decision experts are very limited in this study, and (iii) the considered study focuses on an Indian company. In the future, we will try to evade the drawbacks of this study. In addition, different MCDM methods such as GLDS, DNMA and MAIRCA models can be implemented for DT project risks assessment. The proposed methodology can also be applied to DT project risks assessment in the field of transportation, healthcare management and manufacturing industry. Furthermore, the developed ranking framework can be implemented in numerous risk areas such as strategic assessment, blockchain technology and operations, third party, forensics, cyber, privacy, or any other risk landscape in any digital ecosystem. Based on the applicable risk areas for the digital transformation initiatives, diverse control procedures require to be planned as per important values and organization practices. The critical facet in describing the controls is to take into consideration the type and level of digital transformation in the operations, as most of these regions are at an emerging phase and firmly united with systems or physical procedures, so there might be restrictions to apply the controls. Last but not the least, the developed ranking framework can be implemented to deal with a broader range of project risk assessment problems in the abovementioned areas. These considerations are planned as future research directions.

## Data Availability

All data generated or analysed during this study are included in this published article.
